# Linking adiponectin expression and kidney dysfunction among Indian patients with and without diabetic nephropathy

**DOI:** 10.6026/97320630018876

**Published:** 2022-10-31

**Authors:** Veluri Ganesh, Murugan M, Veerabhadra Goud GK, Vijay Kumar Gangannagari

**Affiliations:** 1 Department of Biochemistry, Aarupadai Veedu Medical College & Hospital, Vinayaka Mission's Research Foundation (Deemed to be University), Salem- 636308, Tamilnadu, India; 2 Department of Biochemistry, Andaman and Nicobar Islands Institute of Medical Sciences, Port Blair - 744104, Andaman and Nicobar Islands, India; 3 Department of Microbiology, Akash Institute of Medical Sciences and Research Centre, Bangalore - 562110, Karnataka, India

**Keywords:** Adiponectin, HbA1c, Microalbumin, Diabetic Kidney Disease

## Abstract

Diabetic kidney disease is a common cause of end stage renal disease has a high incidence rate in population with type 2 diabetes mellitus patients. Adiponectin is an adipocytokine shown to strive anti-diabetic, anti-oxidative as well as anti-inflammatory. The present study aimed to determine the serum adiponectin levels in type 2 diabetes mellitus and explore its association with nephropathy. This cross sectional study recruited 90 type 2 diabetes mellitus patients with (n = 60) and without nephropathy (n = 30). Additionally 30 age, gender, and body mass index matched healthy controls were included. Enzyme linked immunosorbent assay method were used to determine adiponectin concentration. Blood sugars, glycated hemoglobin, body mass index all sounded to be brawny risk factors for nephropathy and microalbumin, e GFR showed a significant association with kidney disease progression in type 2 diabetes mellitus with nephropathy. Both the groups of type 2 diabetes mellitus patients had elevated adiponectin concentrations than controls. Serum adiponectin concentrations were significantly higher in type 2 diabetes mellitus without nephropathy and there was a significant association with nephropathy activity (P<0.0001**). The receiver operating characteristics curve analysis was used to examine the diagnostic performance of adiponectin for nephropathy shown a significant area under the curve 0.998 with sensitivity 100% and specificity 93.33% (P<0.0001**). Hence our study findings concluded that serum adiponectin concentrations considered for the early predictable and prognostic marker for nephropathy.

## Background:

Diabetic kidney disease (DKD) is caused by multiple etiologies, is basically preventable to large extent, and is potentially reversible if diagnosed and treated early. Failure to predict on early stage leads to nephropathy results end stage renal disease (ESRD), usually leads to dysfunction of kidney and associated with high morbidity [1]. Various indices based on biochemical, clinical, and patient-reported variables are in use for the assessment of renal impairment. Among them, the microalbumin is widely used for monitoring of DKD. Management mainly aims at achieving early detection of the disease in order to improve adequate of life and also impede further complications [2]. Amidst being a non specific marker, contemporaneously present in other diseases such as obesity, hypertension, non diabetic patients, cardiovascular diseases and urinary tract infections, furthermore many number of patients with T2DM showed advanced renal pathological events without microalbumin [3]. Large size of albumin than pore size of slit diaphragm of glomerular basement membrane to delay proteinuria. There was a need new and specific diagnostic markers are increasingly being challenged for early prediction of DKD. Adiponectin is a adipocytokine originates in adipose tissue and other tissues like kidney, liver, bones, skeletal muscles, salivary glands [4]. It has 244 amino acids and exists in blood circulation in three different molecular isoforms like high, middle and low molecular weight. The physiological properties of adiponectin involved in anti- diabetic, anti-oxidative and anti-inflammatory actions in patients with T2DM [5]. Adenosine mono phosphate (AMP) protein kinase is a tissue membrane protein get trigger by ADIPO R1 receptor results increase insulin sensitivity, fatty acid activation and glucose up take into tissues [6-8]. Additionally involved in prevention of proteinuria by its reno protective actions through activation of AMP Kinase and NADPH. Circulating adiponectin concentrations are high in metabolic related diseases like T2DM and its complications particularly on kidney [9, 10]. An increase adiponectin activity is a sensitive test for diabetic nephropathy and its progression. There is a need to evaluate the sensitive and specific biomarker than microalbumin to predict early onset of nephropathy and its progression in T2DM patients. However, the present study aimed to investigate whether adiponectin is an earlier predictable marker than for DKD by all the three subgroups of T2DM patients according to microalbumin and also assess the potential roles of adiponectin test the diagnostic indicator of incident nephropathy among individuals on with and without nephropathy as well as controls.

## Materials and Methods:

This cross sectional study conducted in the department of biochemistry, Basaveshwara Institute of Medical Sciences, Karnataka. Patients with T2DM attending the general medicine outpatient clinic fulfilling the American Diabetes Association (ADA) criteria and Kidney Disease Improving Global Outcomes (KIDGO) classification criteria [11, 12]. Subjects with other type’s diabetes, smoking, alcoholism, pregnant women, liver diseases, other kidney diseases, hypertension, cardiovascular, cerebrovascular, thyroid, peripheral vascular diseases were excluded from this study. Ninety patients with T2DM (Normoalbuminuria n = 30, Microalbuminuria n = 30 and Macroalbuminuria n = 30) were enrolled. We had enrolled 30 without nephropathy and 60 with nephropathy for the purpose of exploring the relationship of adiponectin with DKD. Additionally thirty age, gender and BMI matched healthy individuals were enrolled. All the study participants were included in the study after approved by the institutional ethics committee (IEC No: 2018-19/07).

### Sample Collection:

First midstream morning fasting blood sample, urine and post parandial blood sample were collected from all the participants and separated by centrifugation at 3000 rpm for 10 mins. The separated samples were transferred into properly labeled aliquots were stored at -80°c until biochemical analysis was done. Fasting blood sugar (FBS), post parandial blood sugar (PPBS), glycated hemoglobin (HbA1c), urea, creatinine was analyzed by standard methods and microalbumin measured by immunoturbidometric method (EM 200 autoanalyzer). Estimated glomerular filtration rate (e GFR) were calculated modified epic formulae and serum adiponectin was determined by enzyme linked immunosorbent assay (ELISA kit obtained from Biocodon Technologies, Kanasas, USA). The polyclonal antibodies used against adiponectin. Antigens - adiponectin from a serum sample and immobilized adiponectin antigens compete for anti-adiponectin anti-bodies. Streptavidin Anti Horseradish peroxidase (HRP) conjugate is used for detection of bound antibodies. The remaining concentration of antibody conjugate bound to immobilized adiponectin antigens measured photometrically at 450 nm wavelengths. The concentration of adiponectin was read from a standard curve constructed on commercial standards.

### Statistical Analysis:

The data were found to normal distribution by kolmogorov - smrinov test and expressed as mean ± standard deviation (SD). Comparison of data was done by one way analysis of varience (ANOVA) between the T2DM patients and health controls. Turkey post - hoc test was done to analyze statistical significant difference between the groups. Pearson correlation analysis was done in between serum adiponectin and other biochemical parameters. Receiver operating characteristic (ROC) curve analysis was done to arrive at the cutoff levels of serum adiponectin in patients with diabetic nephropathy. All the statistical analysis was performed by using the IBM statistical package for the social sciences (SPSS) version 20.0 and medicalc software. P value < 0.05 was considered statistically significant.

## Results:

### Comparison of baseline, clinical characteristics of T2DM patients and controls:

The baseline and clinical characteristics of the cohort are shown in Table 1(see PDF). The data in our study normally distributed, so the comparison between the patients with T2DM and healthy individuals were performed using ANOVA. We found that the levels of age, FBS, PPBS, urea, creatinine, HbA1c, eGFR, microalbumin and adiponectin in T2DM were significantly higher than those in healthy individuals (P < 0.001*, 0.0001**). The levels of e GFR was significantly decreased in T2DM when compared to healthy individuals (P <0.0001**). In addition, levels BMI no significant difference in between cases and controls (P<0.026).

### Relationship between serum adiponectin and various clinical parameters:

The mean levels of serum adiponectin was proportionately increased from Group 2 to Group 4 (8.96 ± 3.14, 14.93 ± 2.14, 21.63 ± 4.95) when compared to healthy controls (3.22 ± 4.95), there was a statistically significant difference between the groups with (P = 0.0001**). Microalbumin levels were within the normal limits in Group I and II ( 3.42 ± 2.58 mg/dl & 10.10 ± 9.00 mg/dl) and in Group III and IV showed higher levels of microalbumin ( 134.93 ± 74.51 mg/dl & 553.40 ± 182.53 mg/dl) there was a significant difference between all the groups as shown (P = 0.0001**). The eGFR was found to be 91.32 ± 11.89 ml/min and 81.98 ± 24.85 ml/min in Groups I & II respectively and they were within the normal limits with no significant difference between the groups. In Group III and Group IV, eGFR was 67.80 ± 11.66 ml/min and 42.53 ± 8.98 ml/min with the (P = 0.0001**) Table 1 & 2(see PDF).

### Cohort characteristics among the study subjects:

For continuous variables, parameters that followed a normal distribution were analyzed by ANOVA and represented as mean ± SD. All the study subjects were found in the age group of 30 to 70 years. The mean levels of fasting and post parandial blood sugars, HbA1c and adiponectin levels markedly elevated in all the case groups when compared to healthy individuals with the (P = 0.0001**). There was a significantly increased levels of urea, creatinine were observed in Group 3 and Group 4 when compared to Group 1 and 2, respectively (P = 0.0001**). Turkeys post hoc analysis shown no significant difference between age in Group 3 and Group 4 (0.816), BMI in Group 2 when compared to Group 3 and 4 (P = 0.151, 0.075), also Group 1 and Group 4 ( P = 0.985). No significant difference of FBS in Group 2 and Group 3, (P = 0.821), additionally FBS, PPBS is not significant in Group 3 and 4 (P = 0.028, 0.551) Table 3(see PDF).

### Correlation between serum adiponectin, microalbumin, eGFR and other parameters:

Serum adiponectin expression levels were positively correlated with age, FBS, PPBS, HbA1c, Urea, creatinine and microalbumin (r = 0.333, 0.592, 0.622, 0.741, 0.789, 0.762 and 0.771, respectively; all P = 0.0001**) and negatively correlated with eGFR (r = - 0.688, P = 0.0001**). Additionally there were no correlation between the adiponectin with BMI (r = 0.009, P = 0.918). There were negative correlation between eGFR with age, FBS, PPBS, HbA1c, urea, creatinine, microalbumin, adiponectin (r = - 0.341, - 0.581, - 0.457, - 0.639, - 0.650, - 0.662, - 0.697 and – 0.688, respectively; all P = 0.0001**) and no correlation with BMI (r = 0.104, P = 0.259). The microalbumin were positively correlated with age, FBS, PPBS, HbA1c, urea, creatinine and adiponectin (r = 0.456, 0.456, 0.732, 0.741, 0.816 and 0.771; respectively all P = 0.0001**) and negative correlation between BMI and eGFR (r = -0.262 & - 0.697, P = 0.0001**) (Table 4 - see PDF).

## Discussion:

Adipose tissue is the major energy sources in the form of storage, advance lifestyle changes, western food habits, stress, smoking, alcoholism, metabolic and genetic factors are the major risk factors for increase the adiposity in the body leads to obesity [13]. Obesity is the most common factor for to get systemic and metabolic disorders, type 2 diabetes mellitus is also one of the chronic metabolic disorders due to insulin resistance and improper secretion of insulin from the pancreas [14]. Improper functions of insulin and insulin resistance in tissues induced by hyperglycemia results mobilization of fatty acids in the tissues, simultaneously production of free radicals and decreased levels of anti oxidants leads to many micro and macro vascular complications particularly on kidney [15]. In this condition many adipocytokine are produced from adipose tissues, adiponectin is the most abundant adipocytokine secreted from white adipose tissue and also it is secreted from the other tissues like liver, kidney, bones, pancreas, skeletal muscles and some of the glands. The physiological properties of adiponectin play a good role in type 2 diabetes mellitus like insulin sensitization, prevents the damage of certain types of tissues from the oxidants and inflammatory components [16].

In present study, serum adiponectin levels were measured in patients with type 2 diabetes mellitus who were classified further based on the amount of protein excretion into three groups and the levels were compared with age and gender matched healthy controls. Serum adiponectin significantly elevated levels were detected in patients with T2DM in the pre nephropathy stage and it was significantly higher levels were found from normoalbuminuria stage to macroalbuminuria stage when compared to healthy individuals respectively P = 0.0001** (Table 2 - see PDF) (Figure 1 - see PDF). In patients with T2DM with normoalbuminuria group significantly positive correlation to microalbumin as a marker of early predictive and severity of nephropathy. Further analysis using post hoc tests showed that patients with Macroalbuminuria had significantly higher adiponectin levels when compared to other two groups of type 2 diabetes mellitus and controls, respectively; P =0.011. The beneficial actions of adiponectin protect kidney through the activation of AMPK Kinases in podocytes and promotes the normal function of slit diaphragm to maintain pore size in glomerular basement membrane. By this mechanism serum adiponectin prevents the leakage of proteins through the glomerular basement membrane [17]. Similarly previous studies also reported elevated levels of serum adiponectin in type 2 diabetes mellitus to improve the insulin sensitization through this it will control blood sugar levels and it will protects the different tissues damage from the free radicals and inflammatory components through its anti oxidative and anti inflammatory actions [18, 19].

Furthermore, (Table 2 - see PDF) (Figure 1 - see PDF) illustrates there were significantly elevated levels of microalbumin in patients T2DM with microalbuminuria and macroalbuminuria. The microalbumin levels were normal limits in T2DM patients with normoalbuminuria and healthy controls, respectively P = 0.0001**. Additionally we found there were decreased levels of eGFR in group III and IV (67.80 ± 11.66 and 42.53 ± 8.98 but there was a normal limits of eGFR in Group I and healthy individuals (81.98 ± 24.85 and 91.32 ± 11.89) respectively al P values are < 0.0001**. Similarly other recent studies also reported elevated levels of urinary albumin levels in type 2 diabetes mellitus with micro albuminuria when compared to normo and healthy individuals, along with that they reported urinary albuminuria is not an a specific and sensitive biomarker for early detection of nephropathy in type 2 diabetes mellitus due longer duration to excrete due to size of the albumin is more, it is elevated in other conditions like obesity, hypertension, other systemic and metabolic disorders [20].

Correlation of Serum adiponectin with BMI, HbA1c, eGFR and Microalbumin

In the present study, the serum adiponectin is positively correlated with microalbumin, HbA1c and negatively correlated with eGFR, respectively; all P = 0.0001** and there were no significant correlation with BMI (Table 4 - see PDF) (Figure 2 - see PDF). In the patients with nephropathy observed decreased levels of renal clearance along with increased levels of microalbumin when the serum adiponectin are also increased. These study findings supports previous study's and confirmed that the elevated levels of serum adiponectin was identified before excretion of urine albumin, this levels are increased in type 2 diabetes mellitus with normoalbuminuria [21, 22].

Table 5, Figure 3 (see PDF) illustrates receiver operating characteristic (ROC) curves analysis applied to the study results to examine diagnostic performance of serum adiponectin to predict diabetic nephropathy in normoal buminuria cases versus healthy individuals at different cut-off values. There were no significant area under the curve (AUC: 0.647, 0.638) of glycated hemoglobin and microalbumin with the sensitivity and specificity ranging from 37.93 %, 62.07% and 86.67 %, 80.00 %, respectively P values 0.042 and 0.095. The BMI, eGFR showed a significant at area under the curve (0.688, 0.709) sensitivity ranging from 80.00 To 63.33 and specificity from 60.00 To 83.33 with P values < 0.008* and <0.003*. Among these clinical markers of nephropathy serum adiponectin shown statistically highly significant area under the curve (0.998) with sensitivity 100% and specificity 93.33 % respectively P = 0.0001**. This makes serum adiponectin a specific and sensitive early marker as a predictor of nephropathy in T2DM patients with normoal buminuria.

## Conclusions:

It could be concluded that serum adiponectin might be used as a sensitive, specific marker to predict early onset of nephropathy and its progression in patients with type 2 diabetes mellitus.
